# Microbiome Integrity Enhances the Efficacy and Safety of Anticancer Drug

**DOI:** 10.3390/biomedicines13020422

**Published:** 2025-02-10

**Authors:** Alice N. Mafe, Dietrich Büsselberg

**Affiliations:** 1 Department of Biological Sciences, Faculty of Sciences, Taraba State University, Main Campus, Jalingo 660101, Taraba State, Nigeria; mafealice1991@gmail.com; 2 Weill Cornell Medicine-Qatar, Education City, Qatar Foundation, Doha Metropolitan Area, Doha P.O. Box 22104, Qatar

**Keywords:** therapy, microbiome integrity, anticancer drug therapy, drug–microbiome interactions, therapeutic outcomes, cancer treatment safety

## Abstract

The intricate relationship between anticancer drugs and the gut microbiome influences cancer treatment outcomes. This review paper focuses on the role of microbiome integrity in enhancing the efficacy and safety of anticancer drug therapy, emphasizing the pharmacokinetic interactions between anticancer drugs and the gut microbiota. It explores how disruptions to microbiome composition, or dysbiosis, can alter drug metabolism, immune responses, and treatment side effects. By examining the mechanisms of microbiome disruption caused by anticancer drugs, this paper highlights specific case studies of drugs like cyclophosphamide, 5-fluorouracil, and irinotecan, and their impact on microbial diversity and clinical outcomes. The review also discusses microbiome-targeted strategies, including prebiotics, probiotics, postbiotics, and fecal microbiota transplantation (FMT), as promising interventions to enhance cancer treatment. Furthermore, the potential of microbiome profiling in personalizing therapy and integrating these interventions into clinical practice is explored. Finally, this paper proposes future research directions, including developing novel biomarkers and a deeper comprehension of drug–microbiome interactions, to respond to current gaps in knowledge and improve patient outcomes in cancer care.

## 1. Introduction

The gut microbiome is a diverse community of microorganisms in the gastrointestinal tract that plays a vital role in human health and physiological processes [1]. In cancer therapy, interactions between anticancer drugs and the gut microbiome have gained attention, as disruptions in microbiome composition and function can influence drug metabolism, efficacy, and toxicity [2]. Dysbiosis not only compromises gut health but also exacerbates treatment-related side effects, including gastrointestinal toxicity and immune dysregulation [3].

A balanced microbiome supports effective drug metabolism, enhances immune responses, and reduces side effects, highlighting its importance in cancer treatment success [4]. However, antimicrobials used in cancer care may further disrupt the microbiome, complicating therapeutic outcomes [5].

This paper explores strategies to preserve microbiome integrity in cancer therapy, examining drug–microbiome interactions, case studies, and microbiome-targeted interventions to optimize treatment efficacy and safety.

## 2. Methodology

This review synthesized knowledge on the role of microbiome integrity in enhancing the efficacy and safety of anticancer drugs, with data collected from January 2020 to December 2024 to include recent and relevant research. A systematic search was conducted using PubMed, Scopus, and Web of Science, ensuring comprehensive coverage of peer-reviewed studies on microbiome integrity and cancer therapy. Keywords such as “microbiome integrity”, “anticancer drugs”, “drug efficacy”, “gut microbiota”, and “microbiome-targeted interventions” were used, with Boolean operators (and, or, not) to refine the search and exclude irrelevant studies.

Inclusion criteria focused on peer-reviewed English-language articles, research on microbiome–drug interactions, and interventions like probiotics, prebiotics, or fecal microbiota transplantation. Studies published between 2020 and 2024, including clinical trials, case studies, and reviews, were prioritized. Exclusion criteria filtered out non-English publications, non-human studies without translational relevance, unrelated microbiome research, and grey literature. Data from selected studies were thematically analyzed to identify knowledge gaps and highlight future research opportunities, ensuring findings are comprehensive and relevant to advancing cancer treatment strategies.

## 3. Pharmacokinetics of Anticancer Drugs and Microbiome Interactions

The pharmacokinetics of anticancer drugs, encompassing their absorption, distribution, metabolism, and excretion, play a fundamental role in determining their therapeutic efficacy and safety [6]. The gut microbiome has a vast metabolic capacity and significantly influences these pharmacokinetic processes [7]. By modulating drug metabolism, altering bioavailability, and affecting systemic drug levels, the microbiome can directly impact the outcome of cancer therapies [8]. Conversely, anticancer drugs can disrupt the delicate balance of the gut microbiota, leading to dysbiosis and further complicating drug metabolism [9]. This intricate bidirectional interaction marks the need for a deeper awareness of pharmacokinetics in the context of microbiome health, as it holds the potential to optimize treatment strategies and minimize adverse effects in cancer therapy [10]. The intricate interactions between anticancer drugs and the gut microbiota, such as cyclophosphamide, 5-FU, and irinotecan alter microbiome composition and function by reducing microbial diversity and selectively targeting specific bacterial populations. These changes influence drug metabolism, leading to either activation or inactivation of metabolites, and modulate the immune response, potentially impacting treatment efficacy and patient outcomes [11].

### 3.1. Absorption, Distribution, Metabolism, and Excretion (ADME) of Anticancer Drugs and the Role of Microbiota

Absorption: this is the initial step in which a drug enters systemic circulation from its administration site, primarily through the gastrointestinal (GI) tract for oral medications [12]. Drug-specific properties such as solubility, molecular size, and lipophilicity significantly influence absorption [13]. Lipophilic drugs, like tamoxifen and doxorubicin, exhibit better uptake, while poorly soluble drugs, such as paclitaxel, require solubilizers or formulation adjustments [14]. The GI environment, including pH levels, digestive enzyme activity, and gastric emptying rates, also affects absorption, as observed in capecitabine’s pH-dependent activation or irinotecan’s enzymatic conversion to SN-38 [15]. Moreover, the gut microbiota exerts a substantial impact on drug absorption by activating prodrugs (e.g., irinotecan to SN-38) or degrading active drugs (e.g., gemcitabine), thus modulating bioavailability and efficacy [16]. Efflux transporters like P-glycoprotein (P-gp) further regulate absorption by actively expelling drugs from cells, such as paclitaxel and doxorubicin, reducing their systemic availability [17].

Distribution: this involves the transport of drugs from systemic circulation to tissues, influenced by plasma protein binding, tissue permeability, and the drug’s volume of distribution (Vd) [18]. Drugs with high plasma protein binding, such as cisplatin, exhibit reduced free drug availability, affecting therapeutic efficacy. Lipophilic drugs permeate cellular membranes with ease, while hydrophilic drugs rely on active transport mechanisms [19]. The blood–brain barrier (BBB) poses a significant obstacle to central nervous system (CNS) delivery, limiting the distribution of many anticancer drugs [20]. The gut microbiota plays a role in modulating distribution by influencing systemic inflammation and altering plasma protein binding through metabolites like lipopolysaccharides [21]. Also, microbiota-derived short-chain fatty acids (SCFAs) affect BBB integrity. Dysbiosis is characterized by microbiota imbalance that can disrupt these processes, potentially reducing drug availability at target sites [22].

Metabolism: drug metabolism primarily occurs in the liver and involves Phase I (functionalization) and Phase II (conjugation). Phase I reactions are mediated by cytochrome P450 enzymes, oxidize, reduce, or hydrolyze drugs, either activating or inactivating them [23]. For instance, tamoxifen’s therapeutic efficacy depends on its conversion to active metabolites via CYP2D6. Phase II reactions conjugate drugs to enhance solubility and facilitate excretion, as seen in cisplatin detoxification through glutathione-S-transferases [24]. The microbiota significantly contributes to drug metabolism by complementing or competing with hepatic processes. Cyclophosphamide requires microbial activation for immunomodulatory effects, whereas bacterial enzymes inactivate drugs like gemcitabine, diminishing their efficacy [25]. Enterohepatic recirculation is influenced by bacterial β-glucuronidase which prolongs drug half-life, thereby modifying pharmacokinetics [26].

Excretion involves eliminating drugs and their metabolites, predominantly through renal or biliary pathways. Renal excretion involves filtration, secretion, and reabsorption, exemplified by methotrexate, which undergoes these processes in the kidneys [27]. Impaired renal function can lead to drug accumulation and toxicity. Biliary excretion eliminates conjugated drugs like irinotecan’s active metabolite SN-38 via feces. Non-renal pathways, such as exhalation, sweat, and breast milk, serve as minor routes. The microbiota influences excretion by mediating enterohepatic recirculation, which deconjugates bile-excreted drugs, extending their half-life and altering elimination dynamics [28]. Dysbiosis can unpredictably modify these pathways, impacting therapeutic outcomes and toxicity profiles.

Clinical implications: the variability in ADME processes, driven by genetic factors, microbiome composition, and interactions with co-administered drugs, underscores the complexity of optimizing anticancer therapies [29]. The gut microbiota emerges as a critical factor in modulating drug pharmacokinetics, influencing absorption, distribution, metabolism, and excretion [30]. Insight into these microbiota–drug interactions is essential for minimizing adverse effects and enhancing therapeutic efficacy. This highlights the need for integrated research into pharmacokinetics and the microbiome to advance personalized cancer treatment strategies [31].

### 3.2. Impact of Dysbiosis on Drug Metabolism and Efficacy

Dysbiosis is the disruption of the gut microbiota’s balance, which has significant consequences for drug metabolism and efficacy, especially in cancer therapy [32]. The complex interplay between anticancer drugs and the gut microbiota articulates how dysbiosis can alter therapeutic outcomes by modifying drug bioavailability, pharmacokinetics, and patient response [33].

#### 3.2.1. Altered Drug Metabolism

Dysbiosis affects drug metabolism by disrupting microbial enzymatic activity, leading to unpredictable pharmacokinetic profiles:

Reduced drug activation: many prodrugs rely on microbial enzymes for activation [34]. For instance, cyclophosphamide’s immunomodulatory effects depend on gut microbial metabolism [35]. Dysbiosis can impair such activation, reducing therapeutic efficacy [36].

Increased drug degradation: dysbiotic microbiota can degrade active drugs prematurely, thereby lowering their effective concentration [37]. For example, dysbiosis-induced changes in microbial cytidine deaminase activity can accelerate the degradation of gemcitabine, reducing its potency against tumors [38].

Impaired detoxification: the reactivation of detoxified drug metabolites in the gut and is often mediated by microbial β-glucuronidase then altered in dysbiosis [39]. This disruption can lead to either drug accumulation or insufficient drug clearance, exacerbating toxicity [40].

#### 3.2.2. Impact on Drug Efficacy

Dysbiosis alters the therapeutic efficacy of anticancer drugs in several ways:

Diminished tumor-suppressive effects: the gut microbiota produces metabolites like short-chain fatty acids (SCFAs) that modulate the immune system [41]. Dysbiosis reduces SCFA production, impairing immune responses and diminishing the tumor-suppressive effects of drugs like immune checkpoint inhibitors [42].

Resistance development: dysbiosis can induce microbial changes that lead to drug resistance [43]. For instance, alterations in microbial composition can up-regulate efflux transporters or enzymes that degrade anticancer drugs, reducing their effectiveness [44].

#### 3.2.3. Exacerbation of Side Effects

Dysbiosis amplifies the toxicity and side effects of anticancer drugs:

Gastrointestinal toxicity: dysbiosis disrupts the intestinal barrier, increasing the permeability of toxic metabolites [3]. Drugs like irinotecan exacerbate dysbiosis and cause severe diarrhea and gastrointestinal inflammation due to altered microbial β-glucuronidase activity [45].

Immune dysregulation: the microbiota plays a critical role in maintaining immune homeostasis [46]. Dysbiosis-induced immune dysregulation can worsen systemic inflammation, heightening drug-related side effects [47].

#### 3.2.4. Microbiome–Drug Interactions and Antimicrobial Use

The co-administration of antibiotics in cancer patients often worsens dysbiosis, compounding the adverse effects on drug metabolism:

Antibiotic-induced dysbiosis: antibiotics can eliminate beneficial microbes involved in drug metabolism, further reducing the efficacy of immune therapies and cytotoxic drugs [48]. Studies have shown that antibiotics impair the efficacy of immune checkpoint inhibitors by disrupting the microbiota’s ability to prime the immune system [49].

Antimicrobial resistance (AMR): dysbiosis facilitates the proliferation of resistant strains, complicating treatment and reducing the effectiveness of drugs used alongside antimicrobials [50].

#### 3.2.5. Clinical Implications

Variability in patient outcomes: dysbiosis contributes to interpatient variability in drug response, necessitating personalized approaches to cancer therapy [51].

Microbiome restoration strategies interventions like probiotics, prebiotics, and fecal microbiota transplantation (FMT) are being explored to restore microbiota balance and improve drug efficacy [52].

Integration into precision medicine: microbiome profiling could help tailor anticancer therapies by identifying microbial signatures predictive of treatment success or failure [53].

Dysbiosis significantly disrupts drug metabolism and efficacy, emphasizing the importance of maintaining microbiome integrity during cancer therapy. As research advances, integrating microbiome-targeted interventions into treatment regimens could optimize therapeutic outcomes, reduce adverse effects, and enhance patient survival.

## 4. Mechanisms of Microbiome Disruption by Anticancer Drugs

The gut microbiome plays a pivotal role in maintaining physiological homeostasis, yet it is highly susceptible to external stressors, including anticancer drugs [54]. While these drugs are designed to target malignant cells, their systemic effects often extend to the gut microbiota, disrupting its delicate balance [55]. This disruption is known as dysbiosis, which arises through various mechanisms, including direct cytotoxic effects, immune modulation, and interference with microbial metabolic pathways [56]. Such changes can impair gut integrity, reduce microbial diversity, and provoke systemic consequences that influence drug efficacy and patient health [57]. This section delves into the specific mechanisms by which anticancer drugs disrupt the microbiome, exploring the implications for therapeutic outcomes and drawing attention to the need for microbiome-preserving strategies in oncology.

### 4.1. Pathways Through Which Anticancer Drugs Cause Dysbiosis

**Figure 1:**
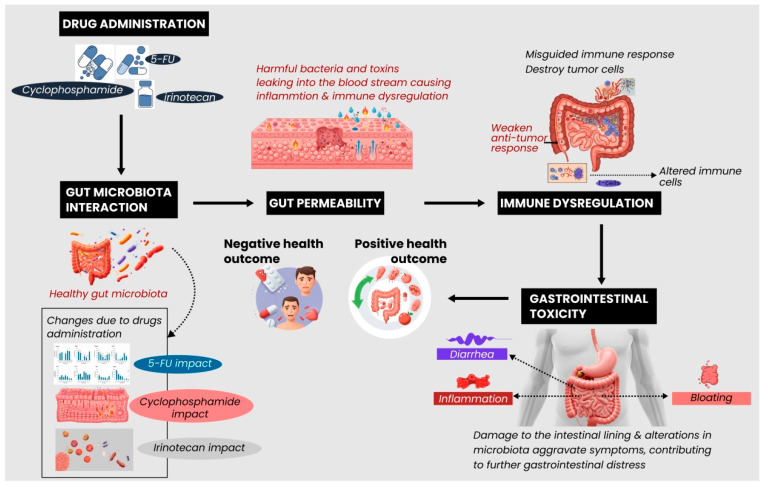
Pathways through which anticancer drugs result in dysbiosis (created on BioRender).

While designed to target and eliminate cancerous cells, anticancer drugs often have unintended consequences on the gut microbiome [58]. The microbiome, a complex community of microorganisms residing in the gastrointestinal tract, plays a crucial role in immune function, metabolism, and overall health [59]. However, many anticancer treatments disrupt this delicate balance, leading to dysbiosis. An imbalance in microbial diversity characterizes dysbiosis and can arise through several pathways, including direct cytotoxic effects, immune modulation, disruption of microbial metabolic processes, and alterations in gut lining integrity [60]. Discerning these pathways is essential for optimizing cancer treatments, as microbiome disruption can impact both the efficacy of anticancer therapies and the overall health of patients [61]. Table 1 highlights how different anticancer drugs interact with the microbiome through various mechanisms, leading to dysbiosis that can influence drug efficacy and overall patient health. Figure 1 illustrates the mechanisms by which anticancer drugs induce dysbiosis, showcasing the impact of chemotherapeutic agents on the gut microbiota. The figure highlights alterations in microbial diversity, decreased beneficial bacteria, and the overgrowth of pathogenic strains, which collectively disrupt the gut microbiome balance [62]. This dysbiosis can lead to systemic effects such as inflammation, compromised gut barrier integrity, and immune modulation, potentially affecting cancer treatment outcomes [63].

### 4.2. Secondary Effects, Such as Immune Dysregulation and Gastrointestinal Toxicity

Anticancer drugs, while targeting malignant cells, often induce secondary effects that can significantly affect patient health and treatment outcomes [71]. Among these, immune dysregulation and gastrointestinal toxicity are two prominent issues linked to cancer therapies [72]. Immune dysregulation can result from the direct or indirect effects of anticancer treatments, leading to alterations in immune cell function and inflammation [73]. This can undermine the body’s ability to fight infections or even lead to immune-mediated side effects [74]. Gastrointestinal toxicity, another common consequence, stems from the impact of anticancer drugs on the gut lining, microbial composition, and digestive functions [75]. Both immune and gastrointestinal disturbances can not only complicate cancer treatment but also diminish the patient’s overall quality of life [76]. Cognizance of these secondary effects is critical for managing and mitigating adverse outcomes, thus ensuring more effective and safer cancer therapies. Table 2 outlines the secondary effects of anticancer drugs on the immune system and gastrointestinal tract, detailing their mechanisms and the resulting impact on patient health.

## 5. Case Studies of Specific Anticancer Drugs and Their Effects on the Microbiome

The impact of anticancer drugs on the gut microbiome is a growing area of research, as the disruption of microbial communities can significantly influence both the effectiveness and side effects of cancer treatments [82]. This section explores the effects of three widely used anticancer agents on the microbiome; cyclophosphamide, 5-fluorouracil (5-FU), and irinotecan. These drugs, each with distinct mechanisms of action, have been shown to induce dysbiosis through various pathways, including direct cytotoxic effects on microbes, alterations in gut permeability, and immune system modulation. By examining case studies of these drugs, a deeper understanding is provided for the knowledge of how chemotherapy agents interact with the gut microbiota, the implications for treatment outcomes, and potential strategies to mitigate these effects to improve patient care. Table 3 highlights the microbial changes observed with cyclophosphamide, 5-FU, and irinotecan, their effects on efficacy and safety, and any interventions used to manage these impacts. The data indicate the importance of managing microbiome health to optimize cancer treatment outcomes.

Case studies on anticancer drugs like cyclophosphamide, 5-fluorouracil (5-FU), and irinotecan highlight the complex relationship between chemotherapy and the gut microbiome. These drugs cause dysbiosis, which affects the effectiveness and safety of cancer treatments. Their impact on microbial communities occurs through cytotoxic effects, immune modulation, and changes in gut permeability. Maintaining a balanced microbiome during cancer treatment is essential to optimize therapeutic outcomes and reduce side effects like gastrointestinal toxicity, immune dysregulation, and infection susceptibility. Disruptions may also alter drug pharmacokinetics, reducing their effectiveness. This review underscores the importance of managing the gut microbiota to enhance cancer treatment and minimize complications. Interventions like probiotics, fecal microbiota transplantation (FMT), and prebiotics show promise in mitigating adverse effects. As research advances, incorporating microbiome health management into cancer care could lead to more personalized and effective treatments, improving patient outcomes. The case studies of cyclophosphamide, 5-FU, and irinotecan emphasize the microbiome’s critical role in cancer therapy, urging further investigation and intervention strategies.

## 6. Role of Antimicrobial Agents in Cancer Therapy

Antimicrobial agents include antibiotics and antifungals that are commonly used in cancer treatment to prevent or treat infections from immunosuppression caused by chemotherapy [86]. While these agents are essential for managing infections, their use can significantly disrupt the gut microbiome, leading to dysbiosis [87]. This dysbiosis can, in turn, impact the efficacy of cancer therapies and exacerbate treatment-related side effects [88]. The balance of gut bacteria plays a crucial role in modulating drug metabolism, immune function, and gastrointestinal health, vital components of effective cancer treatment [89]. This section will explore the complex effects of antibiotics and antifungals on the gut microbiome during cancer therapy, examine how they complicate pharmacokinetics and microbiome health, and suggest guidelines for the judicious use of antimicrobials in cancer patients to minimize adverse effects and optimize therapeutic outcomes (Table 4). Antibiotics and antifungals during cancer treatment disrupt the gut microbiome, reducing microbial diversity, eliminating beneficial microbial species, and leading to the overgrowth of resistant strains [90]. These disruptions can lead to significant downstream effects, including altered drug metabolism, impaired immune responses, and compromised gastrointestinal (GI) health, potentially exacerbating treatment side effects and affecting therapeutic efficacy [91].

### 6.1. Impact of Antimicrobials on Pharmacokinetics and Microbiome Health

The use of antimicrobial agents in cancer therapy introduces additional complexity to drug metabolism and the overall health of the gut microbiome [95]. These drugs can interact with the microbial community in several ways, such as by altering the absorption and metabolism of anticancer drugs or modulating the immune system [96]. These interactions can lead to unpredictable therapeutic outcomes, as the microbiome plays a key role in drug metabolism, immune function, and drug resistance [97]. In particular, antimicrobial agents can disrupt the gut’s microbial balance, potentially diminishing the absorption and efficacy of chemotherapeutic agents [98]. This section will delve into the pharmacokinetic consequences of antimicrobial use in cancer therapy and its potential impact on microbiome health, emphasizing the delicate interplay between these drugs and the microbial environment (Table 5).

### 6.2. Guidelines for Judicious Use of Antimicrobials in Cancer Patients

Given the significant impact that antimicrobial agents can have on the gut microbiome and cancer treatment outcomes, it is critical to use these agents judiciously in cancer patients [103]. The gut microbiota plays a fundamental role in drug metabolism, immune modulation, and gastrointestinal health, all of which can influence the success of cancer therapy [104]. Antibiotics and antifungals must be prescribed based on careful consideration of their potential effects on the microbiome and the overall treatment plan [105]. Overuse or inappropriate use of antimicrobials can lead to the development of resistance, microbiome disruption, and worsened treatment outcomes. This section proposes guidelines for the appropriate use of antimicrobial agents in cancer therapy, focusing on minimizing their negative impact on the microbiome while optimizing treatment effectiveness. These guidelines (Table 6) emphasize the importance of balancing the need for antimicrobial therapy with the preservation of gut microbiome health in cancer patients. A strategic approach to antimicrobial use can help minimize the negative impacts on the microbiome, ultimately improving cancer treatment outcomes and patient quality of life.

Antimicrobial agents, including antibiotics and antifungals, are crucial for managing infections in cancer patients, particularly those undergoing chemotherapy, which causes immunosuppression. However, their use can disrupt the gut microbiome, leading to dysbiosis, which can affect cancer therapy efficacy and worsen side effects like gastrointestinal toxicity and immune dysregulation. The gut microbiota plays a key role in drug metabolism, immune function, and gastrointestinal health, all critical for optimizing cancer treatment. Antibiotics like ciprofloxacin and vancomycin can reduce beneficial bacterial diversity, impair immune responses, and affect chemotherapy drug pharmacokinetics. Antifungals like fluconazole and itraconazole can disrupt the gut’s fungal community, leading to opportunistic pathogen overgrowth and complicating cancer treatment. Antimicrobial agents should be used judiciously to optimize cancer care, minimizing broad-spectrum effects. Targeted therapies, probiotics, and microbiome monitoring are essential for supporting microbiome recovery and reducing adverse effects. Integrating microbiome health and antimicrobial stewardship programs into cancer protocols can help balance infection control with microbiome preservation, improving treatment outcomes and patient quality of life. This section emphasizes the need for careful antimicrobial use to enhance cancer treatment efficacy while minimizing complications.

## 7. Microbiome-Targeted Strategies to Enhance Cancer Treatment

The human microbiome plays a critical role in health and disease, with growing evidence suggesting its substantial impact on cancer treatment outcomes [111]. Dysbiosis has been associated with various cancer therapies, which include chemotherapy and immunotherapy [9]. This has sparked interest in microbiome-targeted strategies as a means to enhance cancer treatment efficacy and mitigate treatment-related side effects [112]. Modulating the microbiome, drug metabolism, immune response, and inflammation, which are all crucial factors in cancer therapy, can potentially be influenced [113]. This section explores the potential of prebiotics, probiotics, and postbiotics to modulate the microbiome to support cancer treatment. Furthermore, it examines the emerging role of fecal microbiota transplantation (FMT) and advances in microbiome-targeted drug delivery systems. When applied appropriately, these strategies can potentially optimize cancer treatment outcomes by restoring microbial balance, enhancing immune function, and improving drug absorption and efficacy.

### 7.1. Prebiotics, Probiotics, and Postbiotics for Microbiome Modulation

The human microbiome is notably the gut microbiota and plays a central role in maintaining health and regulating various physiological processes, which include immune response, metabolism, and drug metabolism [114]. Disruptions to this microbial balance are known as dysbiosis, which has been linked to numerous health conditions, including cancer [115]. As cancer therapies such as chemotherapy and immunotherapy can significantly impact the microbiome, there is growing interest in using microbiome modulation strategies to enhance treatment outcomes [116] (Table 7). Prebiotics, probiotics, and postbiotics are potent tools to modulate the microbiome [117]. Prebiotics are non-digestible food components that stimulate the growth or activity of beneficial microbes, while probiotics are live microorganisms that confer health benefits to the host. On the other hand, postbiotics are the bioactive compounds produced by probiotic bacteria during fermentation, offering a range of benefits, including immune modulation and anti-inflammatory effects [118].

### 7.2. Fecal Microbiota Transplantation (FMT) and Its Emerging Role

Fecal microbiota transplantation (FMT) has emerged as a novel therapeutic approach in the field of microbiome medicine, with significant potential in cancer treatment [122]. FMT involves the transfer of fecal matter from a healthy donor to a recipient, aiming to restore a healthy balance of gut microbiota [123]. This process has already been successfully used in treating *Clostridium difficile* infections and is now being explored for its potential to enhance cancer therapies [124] (Table 8). Cancer treatments like chemotherapy and immunotherapy can disrupt the gut microbiota, leading to dysbiosis, which, in turn, affects drug metabolism, immune function, and gastrointestinal health [125]. Emerging evidence suggests that FMT can help restore microbial diversity, improve immune response, and mitigate some of the adverse effects associated with cancer treatment, such as gastrointestinal toxicity and infections [126].

### 7.3. Advances in Microbiome-Targeted Drug Delivery Systems

Microbiome-targeted drug delivery systems are a groundbreaking innovation that merges the fields of microbiology, pharmacology, and nanotechnology [129]. The gut microbiome plays a pivotal role in the absorption, metabolism, and efficacy of various drugs, including cancer therapeutics [130]. Disruptions to this microbiome can lead to suboptimal drug responses, increased side effects, and altered pharmacokinetics, particularly in cancer patients who are often subjected to intensive treatments such as chemotherapy and immunotherapy [116] (Table 9). Recent advancements in drug delivery systems that specifically target the microbiome hold the potential to overcome these challenges [131]. These systems use the microbiome’s composition and functions to enhance drug delivery to specific sites, such as tumor tissues, while minimizing systemic toxicity [132]. For example, microbiome-responsive nanoparticles and bacteria-based drug delivery vehicles have shown promise in selectively releasing therapeutic agents at tumor sites, improving the precision and effectiveness of cancer therapies [133].

The knowledge of the human microbiome’s role in cancer therapy has led to innovative strategies for enhancing treatment outcomes through microbiome modulation. Prebiotics, probiotics, and postbiotics offer promising ways to restore microbial balance, boost immune response, and mitigate chemotherapy and immunotherapy side effects. These interventions not only improve gastrointestinal health but may also influence drug metabolism, making treatments more effective and tolerable. Fecal microbiota transplantation (FMT) is emerging as a potential solution for patients with severe gastrointestinal issues or infection risks due to microbiome disruption. FMT has shown promise in restoring microbial diversity and improving treatment tolerance, though further research is needed to refine its clinical use. Advances in microbiome-targeted drug delivery systems may also revolutionize cancer treatments by targeting drugs more precisely to tumor sites, enhancing efficacy while minimizing systemic toxicity. Microbiome-based strategies hold great potential for optimizing cancer treatment, reducing side effects, and improving patient quality of life. As research progresses, these approaches may become integral to cancer care.

## 8. Integration into Personalized Medicine

Personalized medicine is revolutionizing healthcare by tailoring treatments to individual characteristics, including genetic, environmental, and lifestyle factors [136]. One of the most promising areas in this evolution is the integration of microbiome profiling into personalized cancer therapy [137]. The human microbiome plays a crucial role in modulating immune responses, drug metabolism, and overall treatment efficacy [138]. Enlightenment on how an individual’s microbiome interacts with cancer therapies can lead to more precise, effective, and safer treatment plans [139]. Microbiome profiling offers the potential to predict how a patient will respond to specific therapies, identify biomarkers for early detection, and provide insights into treatment toxicity [140]. By analyzing the composition and diversity of the microbiome, oncologists can fine-tune anticancer treatments such as chemotherapy, immunotherapy, and targeted therapies to improve patient outcomes. This section explores the potential of microbiome-focused strategies in personalized cancer medicine and highlights case studies where microbiome modulation has led to improved therapeutic responses and reduced side effects.

### 8.1. Microbiome Profiling in Personalizing Anticancer Treatments

This has transformed the landscape of cancer treatment by enabling therapies tailored to individual genetic, environmental, and lifestyle factors [141]. Microbiome profiling is one of this realm’s most innovative and promising tools. The human microbiome, notably, the gut microbiota, has emerged as a critical player in influencing the effectiveness of cancer treatments [142]. Research has shown that the composition and diversity of the microbiome can affect drug metabolism, immune responses, and the overall health of cancer patients, influencing their response to therapies [143] (Table 10). Microbiome profiling involves analyzing the microbial community within a patient’s body to gain insights into its composition, diversity, and functionality [144]. By intuition of how specific microbiome profiles interact with cancer therapies, clinicians can potentially predict treatment responses, optimize drug selection, and minimize adverse side effects [145]. This personalized approach aims to provide more effective and safer cancer treatments, ensuring that each patient receives the most suitable therapeutic regimen based on their unique microbiome characteristics [146].

### 8.2. Examples Where Microbiome-Focused Approaches Improved Outcomes

As the proficiency of the gut microbiome’s role in cancer therapy deepens, microbiome-focused approaches have shown promise in improving cancer treatment outcomes [150]. These strategies involve modulating the microbiome through prebiotics, probiotics, fecal microbiota transplantation (FMT), and other interventions to enhance the efficacy of anticancer therapies, reduce treatment-related side effects, and improve patient quality of life [151]. Clinical case studies have highlighted significant improvements in cancer outcomes when microbiome-focused strategies are applied [152]. For instance, restoring microbial diversity through FMT has been linked to enhanced chemotherapy response, while probiotic supplementation has helped alleviate common chemotherapy-induced side effects like gastrointestinal toxicity. Also, microbiome modulation has been shown to impact immune system function, which is crucial for therapies like immunotherapy. This section will present various examples where microbiome-focused interventions have led to improved treatment outcomes, shedding light on the potential of these approaches in the future of cancer care (Table 11).

Integrating microbiome profiling into personalized cancer therapy is an exciting frontier in oncology, with growing evidence that the gut microbiome influences treatment outcomes; tailoring therapies based on an individual’s microbiome could lead to safer and more effective plans. This approach may reduce side effects, enhance chemotherapy efficacy, and improve patient outcomes. Microbiome profiling helps identify the best treatments by analyzing the specific microbial community in a patient’s body. Some microbial populations may enhance treatment effectiveness, while others may reduce responses or increase adverse effects. Cognizance of these interactions allows clinicians to optimize strategies for better results and fewer complications. Case studies show that microbiome-focused strategies like fecal microbiota transplantation (FMT) and probiotics can restore microbial diversity, modulate the immune system, and improve drug metabolism, enhancing treatment efficacy. As research progresses, microbiome profiling is expected to become integral to cancer care, enabling microbiome-guided treatment plans that optimize drug metabolism, immune function, and response. Ultimately, this will lead to more individualized, effective, and patient-centered therapies, improving survival rates and quality of life.

## 9. Public Health and Clinical Implications

The growing body of evidence supporting the role of the microbiome in cancer treatment has profound public health and clinical implications [156]. Microbiome-focused interventions, such as prebiotics, probiotics, fecal microbiota transplantation (FMT), and microbiome-targeted drug delivery systems, have the potential to revolutionize cancer therapy by enhancing the efficacy of treatments, reducing side effects, and improving patient outcomes [157]. As these interventions move from the research stage into clinical practice, evaluating how they can be integrated into routine cancer care becomes essential. Integrating microbiome-focused therapies into clinical practice involves grasping the scientific mechanisms behind these treatments and resolving logistical, economic, and policy-related challenges [158]. In particular, issues such as the standardization of microbiome interventions, training healthcare providers, and ensuring the cost-effectiveness of these therapies need to be carefully considered. This section will discuss the broader implications of microbiome-based strategies for cancer treatment, exploring their potential for improving public health outcomes and examining the challenges in their clinical implementation. Furthermore, it will propose ways to resolve these challenges and ensure that microbiome-based interventions can effectively and equitably integrate into clinical practice.

### 9.1. Integration of Microbiome-Focused Interventions in Clinical Practice

The integration of microbiome-focused interventions into clinical practice represents a transformative approach to cancer treatment, harnessing the intricate relationship between the human microbiome and disease progression [159]. As research continues to uncover the pivotal role of the microbiome in modulating cancer therapy outcomes, interventions such as probiotics, prebiotics, fecal microbiota transplantation (FMT), and microbiome-targeted drug delivery systems have shown promise in enhancing the efficacy of conventional treatments, improving patient quality of life, and reducing treatment-related side effects [160]. However, transitioning these novel therapies from experimental settings to routine clinical care requires overcoming significant scientific, logistical, and regulatory hurdles [161]. In clinical practice, the successful integration of microbiome-based interventions demands not only scientific validation and clinical trials but also careful consideration of practical aspects, including cost-effectiveness, patient access, and the establishment of standardized protocols [162]. Furthermore, it involves educating healthcare professionals on the benefits and potential risks associated with microbiome modulation and incorporating microbiome profiling into personalized treatment plans. This will explore the potential of microbiome-focused interventions in clinical oncology, spotlighting the steps needed to incorporate them into mainstream cancer care and handling the challenges that must be overcome to make these interventions widely accessible to patients. Several case studies have demonstrated the successful integration of microbiome-focused interventions in clinical practice (Table 12).

### 9.2. Economic and Policy-Related Challenges in Implementing Microbiome-Based Strategies

The potential benefits of microbiome-based interventions in cancer therapy are substantial, yet their widespread adoption faces significant economic and policy-related challenges [167]. One of the main obstacles is the high cost associated with developing and implementing microbiome therapies, including the cost of advanced diagnostics (such as microbiome profiling) and personalized interventions like FMT or probiotics [168]. Likewise, healthcare systems and insurance companies can struggle to incorporate these novel therapies into reimbursement frameworks, especially in resource-limited settings [169]. Moreover, there are regulatory challenges regarding the approval and standardization of microbiome-based treatments [170]. As these therapies are relatively new, regulatory agencies such as the FDA or EMA lack clear guidelines, which can delay their clinical adoption. Policymakers also face the challenge of ensuring equitable access to these interventions across diverse populations, as socioeconomic factors can create disparities in access to cutting-edge cancer therapies. The implementation of microbiome-based strategies faces several economic and policy challenges, along with potential solutions, as summarized in Table 13.

Integrating microbiome-focused interventions into clinical practice could revolutionize cancer treatment by enhancing outcomes and reducing side effects through personalized care. However, challenges such as the high cost of microbiome profiling, lack of standardized protocols, regulatory approval, and limited accessibility in low-resource settings hinder broader implementation. Overcoming these barriers requires policy reforms, research and infrastructure investment, and collaboration among healthcare, research, and industry stakeholders to ensure equitable access to microbiome-based therapies and their integration into routine cancer care.

## 10. Future Research Directions

As the recognition of the microbiome’s role in cancer therapy advances, there is a growing need for targeted research to optimize and personalize treatments [174]. The future of cancer treatment will likely involve an integrative approach where microbiome profiling is an essential component of personalized medicine [175]. Research in this area is poised to identify innovative biomarkers for assessing microbiome health in cancer patients, providing a more tailored approach to cancer therapies [176]. Additionally, there is a pressing need to deal with the underexplored interactions between drugs and the microbiome, particularly in how these relationships affect drug efficacy and toxicity [177]. By identifying these interactions, we can reduce adverse side effects, improve therapeutic outcomes, and create more effective strategies for microbiome modulation. This section will explore the key areas for future research, focusing on biomarkers and drug–microbiome interactions, which will further bridge gaps in our cognition and foster new clinical applications.

### 10.1. Proposed Areas for Future Studies

Novel Biomarkers for Microbiome Health in Cancer Patients: The identification of specific biomarkers associated with microbiome health holds great promise for predicting cancer treatment responses, managing side effects, and personalizing therapy [178]. These biomarkers could be used to assess the functional state of the microbiome and its influence on various aspects of cancer therapy, including drug metabolism, immune modulation, and gastrointestinal function [179] (Table 14).

Drug–Microbiome Interactions: Knowledge of the interactions between cancer therapies and the microbiome is crucial for optimizing treatment regimens. The complex relationships between drugs, microbiota composition, and their metabolic byproducts can influence drug efficacy, side effects, and resistance mechanisms [184]. Further research in this area is needed to untangle these interactions and identify strategies for improving drug efficacy and reducing toxicity (Table 15).

### 10.2. Unmet Needs in Perception of Drug–Microbiome Interactions

Although significant progress has been made in recognizing the impact of the microbiome on cancer therapy, several critical gaps remain [188]. Key unmet needs in the perception of drug-microbiome interactions include the following:

Characterization of cancer-specific microbiomes; each cancer type can have a distinct microbiome profile that interacts differently with therapeutic modalities. More research is needed to map these cancer-specific microbiomes and their effects on treatment outcomes [189].

Mechanisms of microbiome influence drug resistance: the microbiome can play a significant role in cancer drug resistance, particularly through modulating the immune system or altering drug metabolism. Further studies are necessary to understand how microbiota affect the efficacy of chemotherapy, targeted therapies, and immunotherapy [82].

Microbiome-based therapeutics in combination with cancer treatments; there is an urgent need for studies evaluating the synergy between microbiome-based interventions, such as probiotics, prebiotics, or fecal microbiota transplantation (FMT), and standard cancer therapies. This will help clarify the potential role of microbiome modulation in improving cancer treatment outcomes [4].

Long-term impact of microbiome interventions: grasping the long-term safety and efficacy of microbiome interventions, especially in combination with cancer treatments, is critical for future research. This includes evaluating the potential risks of altering the microbiome for extended periods [157].

Personalized microbiome interventions: research should focus on how microbiome-targeted strategies can be personalized based on individual microbiome profiles. This approach could be tailored to optimize therapy while minimizing side effects and drug resistance [190].

By focusing on these areas, future studies can lead to innovative approaches in microbiome-based precision medicine, helping to transform cancer care and improve patient outcomes.

## 11. Conclusions

Integrating microbiome-focused strategies into cancer therapy can significantly improve treatment outcomes and patient well-being. The microbiome plays a crucial role in drug metabolism, immune modulation, and treatment efficacy. Microbiome-targeted interventions, such as probiotics, prebiotics, and fecal microbiota transplantation, can optimize therapeutic strategies. Prioritizing microbiome health is essential for advancing personalized medicine, enhancing treatment effectiveness, and minimizing side effects. Future research should focus on uncovering drug–microbiome interactions and identifying novel biomarkers to advance precision oncology.

## Figures and Tables

**Table 1:** Key pathways through which anticancer drugs cause dysbiosis.

Mechanism	Anticancer Drug(s)	Pathway/Effect	Impact on Microbiome
Direct Cytotoxicity	Chemotherapy agents (e.g., 5-FU, cyclophosphamide, doxorubicin)	Kill both cancer cells and gut microbes, especially rapidly dividing ones	Reduction in microbial diversity, loss of beneficial microbes [64]
Immune Modulation	Immune checkpoint inhibitors (e.g., pembrolizumab, nivolumab)	Alter immune responses, promoting inflammation that can damage the gut microbiota	Disruption of microbial balance and increased gut inflammation [65]
Microbial Metabolism Disruption	Antibiotics used in cancer treatment (e.g., broad-spectrum antibiotics like ciprofloxacin)	Alter microbial enzymatic activities, inhibiting drug metabolism and leading to imbalanced microbial communities	Impaired drug metabolism, resistance development, and loss of beneficial bacteria [66]
Antioxidant Activity Interference	Platinum-based drugs (e.g., cisplatin, carboplatin)	Alter oxidative stress pathways, affecting microbial survival and composition	Disturbance in microbial communities decreased abundance of beneficial microbes [67]
Microbial Nutrient Competition	Targeted therapies (e.g., tyrosine kinase inhibitors like imatinib)	Disrupt nutrient availability and microbial–host interactions	Changes in microbial composition, favoring pathogenic or opportunistic bacteria [68]
Damage to Gut Lining	Chemotherapy and radiotherapy (e.g., 5-FU, doxorubicin)	Damage to the gut epithelial cells, allowing pathogenic microbes to proliferate	Increased gut permeability, microbial translocation, exacerbation of dysbiosis [69]
Disruption of Short-Chain Fatty Acid (SCFA) Production	Cyclophosphamide, 5-FU	Impact on the production of SCFAs, which regulate immune function and gut health	Reduced SCFA production, leading to weakened immune responses and compromised gut health [70]

**Table 2:** Secondary effects of anticancer drugs, such as immune dysregulation and gastrointestinal toxicity.

Secondary Effect	Anticancer Drug(s)	Pathway/Mechanism	Impact on Health
Immune Dysregulation	Chemotherapy (e.g., cyclophosphamide, 5-FU), Immunotherapy (e.g., PD-1/PD-L1 inhibitors)	Suppression or activation of immune responses, altering immune cell functions, or causing inflammation	Increased risk of infections, autoimmune disorders, immune-related adverse events (irAEs) [77]
Gastrointestinal Toxicity	Chemotherapy (e.g., 5-FU, doxorubicin, irinotecan), Radiotherapy, Targeted therapies (e.g., EGFR inhibitors)	Direct damage to the gastrointestinal lining, disruption of microbial balance, changes in gut motility	Nausea, vomiting, diarrhea, mucositis, dysbiosis, malabsorption [72]
Chronic Inflammation	Immune checkpoint inhibitors (e.g., pembrolizumab, nivolumab), Targeted therapies	Chronic activation of inflammatory pathways, leading to persistent inflammatory responses	Persistent gut inflammation, worsening side effects, and discomfort [78]
Altered Gut Microbiota	Broad-spectrum antibiotics, Chemotherapy, Targeted therapies	Disruption of microbial diversity, reduction in beneficial bacteria	Dysbiosis, impaired immune function, exacerbation of gastrointestinal symptoms [79]
Mucosal Damage	Chemotherapy (e.g., methotrexate, 5-FU), Radiation therapy	Damage to the mucosal lining of the gut, reduced epithelial cell regeneration	Painful ulcers, increased susceptibility to infections, compromised absorption of nutrients [80]
Impaired Drug Metabolism	Chemotherapy, Antibiotics (used during cancer treatment)	Alteration of microbiome-mediated drug metabolism, affecting anticancer drug efficacy	Reduced effectiveness of treatment, the potential for higher drug toxicity or resistance [81]

**Table 3:** Findings from experimental studies on cyclophosphamide, 5-FU, and irinotecan, including microbial changes, impact on efficacy/safety, and any interventions used.

Drug Name	Microbial Changes Observed	Impact on Efficacy/Safety	Interventions Used
Cyclophosphamide	Reduced diversity of gut microbiota. Decreased abundance of beneficial bacteria (e.g., *Lactobacillus*, *Bifidobacterium*). Increased abundance of pathogenic bacteria (e.g., *Clostridium difficile*).	Increased susceptibility to infections (e.g., *C*. *difficile*). Potential immune suppression, leading to impaired therapeutic response. Altered drug metabolism due to microbiome disruption.	Administration of probiotics (e.g., *Lactobacillus* strains). Use of antibiotics to prevent or treat infections (e.g., Vancomycin for *C. difficile*). Fecal microbiota transplantation (FMT) in certain clinical settings [83].
5-Fluorouracil (5-FU)	Decreased diversity of gut microbiota. Alterations in the abundance of *Firmicutes* and *Bacteroidetes* phyla. Increased abundance of pathogenic bacteria such as *Escherichia coli* and *Enterococcus faecalis.*	Increased gastrointestinal toxicity (e.g., nausea, diarrhea, mucositis). Potential alteration in the metabolism of 5-FU, reducing its therapeutic efficacy. Immune dysregulation due to microbiome imbalance.	Administration of fecal microbiota transplantation (FMT). Probiotic supplementation to restore beneficial microbes (e.g., *Lactobacillus*, *Bifidobacterium*). Use of prebiotics to support beneficial microbiota growth [84].
Irinotecan	Decreased abundance of beneficial microbes, such as *Bacteroides* and *Firmicutes.* Increased levels of pathobionts, including *Enterococcus* and *Clostridium.* Dysbiosis associated with gut inflammation.	Increased gastrointestinal toxicity, particularly diarrhea and colitis. Potential impact on irinotecan metabolism, reducing its therapeutic effectiveness. Immunosuppression leading to increased infection risk.	Administration of antibiotics (e.g., Metronidazole, Vancomycin) to treat or prevent infections. Probiotic supplementation (e.g., *Lactobacillus* strains) to restore gut microbiome balance Dietary modifications to support gut health [85].

**Table 4:** Impact of antibiotics on gut microbiome and cancer therapy.

Antimicrobial Agent	Microbial Changes Observed	Impact on Efficacy/Safety	Interventions Used
Ciprofloxacin	Reduced diversity of gut microbiota, decrease in beneficial bacteria like *Lactobacillus* and *Bifidobacterium*	Increased risk of gastrointestinal toxicity and infections (e.g., *Clostridium difficile*). Potential alteration in anticancer drug metabolism.	Probiotics (e.g., *Lactobacillus*), fecal microbiota transplantation (FMT), and antibiotic stewardship [92].
Vancomycin	Disruption of *Firmicutes* and *Bacteroidetes*, increased abundance of Enterococcus and *Clostridium* species	Increased susceptibility to secondary infections like *C. difficile*, altered drug metabolism, and immune dysregulation.	Probiotics, targeted antibiotic use, monitoring for gut-related infections [93].
Meropenem	Drastic reduction in microbial diversity, loss of beneficial anaerobes	Compromised immune response, impaired chemotherapy efficacy, risk of secondary infections.	Judicious antibiotic use, prophylactic antifungals, gut microbiome restoration through probiotics [94].

**Table 5:** Impact of antifungals on gut microbiome and cancer therapy.

Antimicrobial Agent	Microbial Changes Observed	Impact on Efficacy/Safety	Interventions Used
Fluconazole	Decreased abundance of beneficial fungi like *Saccharomyces cerevisiae*; Increase in opportunistic pathogens [99]	Increased risk of fungal infections and potential interference with chemotherapy drug metabolism.	Antifungal stewardship, probiotics to replenish beneficial microbiota [100].
Itraconazole	Altered gut microbial composition, reduced fungal diversity	Alteration in pharmacokinetics, including potential interactions with anticancer drug metabolism.	Monitoring antifungal levels and reducing use to necessary periods [101].
Amphotericin B	Severe reduction in gut microbial diversity	Potential systemic toxicity and increased susceptibility to secondary infections, including fungal overgrowth.	Use of alternative antifungals with lower systemic toxicity, probiotics [102].

**Table 6:** Guidelines for judicious use of antimicrobials in cancer therapy.

Guideline	Rationale	Recommendations
Targeted antimicrobial therapy	Reduces broad-spectrum impact on gut microbiota and minimizes resistance development.	Use narrow-spectrum antibiotics when possible. Avoid over-prescribing [106].
Probiotic supplementation	Supports microbiome recovery and reduces adverse gastrointestinal effects.	Administer probiotics with evidence for efficacy (e.g., *Lactobacillus* strains) [107].
Regular microbiome monitoring	Ensures early detection of dysbiosis and microbiome imbalances.	Monitor gut microbiome diversity before, during, and after antimicrobial treatment [108].
Incorporating microbiome health into cancer care	Optimizes therapeutic outcomes by maintaining a balanced microbiome.	Integrate microbiome management strategies into cancer care protocols [109].
Antimicrobial stewardship programs	Improves clinical outcomes and reduces unnecessary antimicrobial use.	Implement stewardship programs to guide appropriate antimicrobial use [110].

**Table 7:** Use of prebiotics, probiotics, and postbiotics in cancer treatment.

Strategy	Microbial Changes Observed	Impact on Cancer Treatment	Interventions and Outcomes
Prebiotics (e.g., inulin)	Increase in beneficial microbes like *Bifidobacterium* and *Lactobacillus*	Enhanced immune response, improved gastrointestinal health, reduced chemotherapy-induced toxicity	Increased effectiveness of chemotherapy and reduced side effects like diarrhea and mucositis [119]
Probiotics (e.g., *Lactobacillus*, *Bifidobacterium*)	Restoration of gut microbiota diversity, reduction in pathogenic bacteria	Enhanced drug metabolism, modulation of immune response, reduced inflammation	Reduced frequency of infections, improved immune function, potential for enhanced immunotherapy responses [120]
Postbiotics (e.g., short-chain fatty acids)	Production of metabolites like butyrate that promote gut health	Enhanced gut barrier function, modulation of immune responses, and potential anti-cancer effects	Improvement in gut health, reduced inflammation, and enhanced efficacy of chemotherapy and immunotherapy [121]

**Table 8:** Fecal microbiota transplantation (FMT) in cancer treatment.

Case Study Description	Microbial Changes Observed	Impact on Treatment	Interventions and Outcomes
FMT in Chemotherapy Patients	Restoration of microbial diversity, enrichment of anti-inflammatory bacteria	Reduced chemotherapy-induced gut toxicity, improved immune function	Improvement in chemotherapy tolerance, reduced treatment-related infections and complications [127]
FMT in Immunotherapy Patients	Alteration of microbiome composition, increase in beneficial microbes	Enhanced immune checkpoint inhibitor responses, reduced immunotherapy-related adverse events	Improved overall survival, reduced side effects like colitis [128]

**Table 9:** Microbiome-targeted drug delivery systems.

Technology	Microbial Changes Observed	Impact on Cancer Treatment	Interventions and Outcomes
Microbiome-responsive nanoparticles	Targeted release of drugs based on microbiome composition	Enhanced drug bioavailability, targeted drug release to tumor sites, reduced systemic toxicity	Improved efficacy of chemotherapy and immunotherapy with minimized side effects [134]
Bacteria-based drug delivery systems	Modification of gut microbiota, specific targeting of tumor sites	Increased drug accumulation at tumor sites, enhanced tumor-killing effects	Higher therapeutic efficacy, reduced off-target toxicity [135]

**Table 10:** Microbiome profiling for personalized cancer treatments.

Case Study Description	Microbial Changes Observed	Personalized Treatment Adjustments	Outcomes and Impact
Chemotherapy Response in Colon Cancer	Higher abundance of *Firmicutes* and lower levels of *Bacteroidetes*	Chemotherapy regimen adjusted based on microbiome composition to improve drug efficacy	Enhanced chemotherapy response, reduced gastrointestinal toxicity [147]
Immunotherapy in Melanoma Patients	Increased abundance of certain bacteria (e.g., *Bifidobacterium*)	Immunotherapy adjusted by incorporating microbiome data to enhance response to checkpoint inhibitors	Improved tumor shrinkage, reduced side effects like colitis and diarrhea [148]
Breast Cancer Treatment	Microbiome diversity alterations due to chemotherapy	Probiotic supplementation based on microbiome profile to restore beneficial microbes	Increased chemotherapy tolerance, enhanced immune response, reduced infections [149]

**Table 11:** Examples of microbiome-focused approaches improving cancer outcomes.

Case Study Description	Microbial Modulation Strategy	Outcome	Mechanisms Involved
FMT in Colorectal Cancer Patients	Fecal microbiota transplantation (FMT) to restore microbial diversity	Improved chemotherapy tolerance and response	Restoration of gut microbial diversity, reduced toxicity, improved immune function [153]
Probiotics in Chemotherapy-Related GI Toxicity	Probiotic supplementation with *Lactobacillus* strains	Reduced incidence of chemotherapy-induced diarrhea and mucositis	Restoration of beneficial microbiota, enhanced gut barrier function [154]
Prebiotics in Breast Cancer	Administration of prebiotic fiber (e.g., inulin)	Improved chemotherapy efficacy, reduced side effects	Enhanced beneficial gut bacteria, modulation of immune response, and drug metabolism [155]

**Table 12:** Case studies on the integration of microbiome-focused interventions in clinical practice.

Case Study	Microbiome-Focused Intervention	Clinical Outcome	Challenges	Solutions/Redress
FMT in Colorectal Cancer Patients	Fecal microbiota transplantation (FMT) to restore microbial diversity in patients undergoing chemotherapy for colorectal cancer	Improved chemotherapy efficacy and reduced incidence of gastrointestinal side effects	Lack of standardized protocols for FMT administration, limited availability of donor material	Development of standardized FMT protocols, establishment of microbiome biobanks, and greater investment in research for FMT optimization [163]
Probiotic Supplementation in Breast Cancer Patients	Use of *Lactobacillus* strains to mitigate chemotherapy-induced diarrhea in breast cancer patients	Significant reduction in diarrhea incidence and improved quality of life during chemotherapy	Concerns about long-term safety and efficacy of probiotics in cancer patients	Long-term clinical trials to assess safety, collaboration with regulatory bodies for probiotic safety standards [164]
Microbiome Profiling in Lung Cancer Treatment	Microbiome profiling to predict patient response to immunotherapy	Enhanced response to immunotherapy in patients with diverse microbiomes	High cost of microbiome profiling, need for personalized data integration	Increased funding for microbiome profiling research, collaboration between oncology and microbiome researchers to integrate profiling into clinical workflows [165]
Use of Prebiotics in Pancreatic Cancer Therapy	Prebiotic supplementation to improve gut microbiome diversity in pancreatic cancer patients	Improved immune system response and reduced cancer progression	Limited patient access to prebiotic interventions, challenges in patient adherence	Public health campaigns to raise awareness, subsidized prebiotic access programs in healthcare system [166]

**Table 13:** Economic and policy challenges and solutions for implementing microbiome-based strategies.

Challenge	Description	Proposed Solutions
Cost of Microbiome Profiling	High cost of microbiome analysis (e.g., 16S rRNA sequencing, whole genome sequencing) limits accessibility for patients	Increase funding for research to reduce profiling costs, collaborate with healthcare systems to integrate cost-effective microbiome diagnostics into routine care [171]
Regulatory Uncertainty	Lack of clear regulatory frameworks for the approval of microbiome-based treatments, such as FMT and probiotics, can delay clinical adoption	Work with regulatory bodies to develop clear guidelines for microbiome interventions, create standardized protocols for FMT, and ensure probiotics are evaluated for safety and efficacy in cancer patients
Insurance and Reimbursement Issues	Insurance companies can not cover microbiome-based therapies, making them unaffordable for many patients	Advocate for policy changes that ensure insurance coverage for microbiome-based treatments, engage in discussions with healthcare providers to include microbiome-based therapies in treatment protocols [172]
Equitable Access	Disparities in access to microbiome-based therapies across different socioeconomic and geographical groups	Implement government subsidies or partnerships with pharmaceutical companies to provide affordable access to microbiome therapies, launch public health campaigns to raise awareness and ensure equitable distribution [173]

**Table 14:** Potential biomarkers for microbiome-driven cancer treatment outcomes.

Research Area	Potential Biomarkers	Application	Expected Outcome
Gut Microbiome Composition	Microbial diversity indices, specific beneficial bacteria (e.g., *Lactobacillus*, *Bifidobacterium*)	Predict response to chemotherapy and immunotherapy by assessing microbial composition changes.	Identifying microbiome signatures that predict treatment response and side effects in cancer patients [180].
Metabolic Markers	Short-chain fatty acids (SCFAs), bile acids	Understand the role of microbiome-derived metabolites [181] in modulating immune response and drug efficacy.	Development of markers that assess the metabolic contributions of the microbiome to cancer therapy outcomes [182].
Immune Modulation Signals	Cytokine profiles, T-cell markers	Identify microbiome-driven immune modulation in cancer immunotherapy.	Insights into how specific microbiome profiles influence cancer treatment efficacy through immune pathways [183].

**Table 15:** Research areas in drug–microbiome interactions and cancer therapy.

Research Area	Key Questions	Methods/Approaches	Potential Impact
Impact of Chemotherapy on Microbiome	How does chemotherapy alter microbiome composition and function?	Longitudinal microbiome analysis in cancer patients during chemotherapy.	Identifying chemotherapy-induced microbiome shifts and their correlation with side effects or efficacy [185].
Drug Metabolism and Microbiome	How does the microbiome influence drug metabolism and pharmacokinetics?	Study of microbiome and drug interaction in vitro and in vivo, focusing on metabolic enzymes.	Development of strategies to modulate the microbiome to optimize drug absorption and metabolism [186].
Antibiotics and Cancer Therapy	How do antibiotics used in cancer care affect the microbiome and therapy outcomes?	Comparative analysis of microbiome diversity before and after antibiotic treatment in cancer patients.	Potential to reduce the use of broad-spectrum antibiotics to prevent microbiome disruption and improve outcomes [187].

## Data Availability

No new data were created or analyzed in this study. Data sharing is not applicable to this article.
